# Increased salt tolerance with overexpression of cation/proton antiporter 1 genes: a meta‐analysis

**DOI:** 10.1111/pbi.12599

**Published:** 2016-09-06

**Authors:** Yuan‐Chun Ma, Robert M. Augé, Chao Dong, Zong‐Ming (Max) Cheng

**Affiliations:** ^1^ Institute of Horticulture Jiangsu Academy of Agricultural Sciences/Jiangsu Key Laboratory for Horticultural Crop Genetic Improvement Nanjing Jiangsu China; ^2^ College of Horticulture Nanjing Agricultural University Nanjing Jiangsu China; ^3^ Department of Plant Sciences University of Tennessee Knoxville TN USA

**Keywords:** antiporter, *CPA1* gene family, meta‐analysis, overexpression, salt tolerance, transgenic

## Abstract

Cation/proton antiporter 1 (*CPA1*) genes encode cellular Na^+^/H^+^ exchanger proteins, which act to adjust ionic balance. Overexpression of *CPA1s* can improve plant performance under salt stress. However, the diversified roles of the *CPA1* family and the various parameters used in evaluating transgenic plants over‐expressing *CPA1*s make it challenging to assess the complex functions of *CPA1s* and their physiological mechanisms in salt tolerance. Using meta‐analysis, we determined how overexpression of *CPA1s* has influenced several plant characteristics involved in response and resilience to NaCl stress. We also evaluated experimental variables that favour or reduce *CPA1* effects in transgenic plants. Viewed across studies, overexpression of *CPA1*s has increased the magnitude of 10 of the 19 plant characteristics examined, by 25% or more. Among the ten moderating variables, several had substantial impacts on the extent of *CPA1* influence: type of culture media, donor and recipient type and genus, and gene family. Genes from monocotyledonous plants stimulated root K^+^, root K^+^/Na^+^, total chlorophyll, total dry weight and root length much more than genes from dicotyledonous species. Genes transformed to or from *Arabidopsis* have led to smaller *CPA1*‐induced increases in plant characteristics than genes transferred to or from other genera. Heterogeneous expression of *CPA1s* led to greater increases in leaf chlorophyll and root length than homologous expression. These findings should help guide future investigations into the function of *CPA1s* in plant salt tolerance and the use of genetic engineering for breeding of resistance.

## Introduction

Soil salinization is one of the chief abiotic stresses that constrain agriculture worldwide (Cramer *et al*., 2011), and at least 50% of global agricultural lands are at risk of salinization (Wang *et al*., 2003). Salinity impacts plants by causing osmotic stress, imbalance of ions, ion toxicity and excessive reactive oxygen species (Ruíz‐Lozano *et al*., 2012). Understanding the molecular mechanisms of salt stress responses and the functions of genes that regulate responses to salinity will help in designing strategies for development of salt‐resistant crop plants.

The most extensively studied gene category in relation to NaCl stress physiology is the family of cation/proton antiporter 1 (*CPA1*, Brett *et al*., 2005). The *CPA1* gene family belongs to the *CPA* superfamily and includes the *NHX* type and *Nhap* type (Apse *et al*., 1999; Rodríguez *et al*., 2009). *CPA1* genes encode Na^+^/H^+^ exchanger proteins, which modulate ion balance in plant cells (Feki *et al*., 2014; Fukuda *et al*., 2004). Many *CPA1* genes have been isolated from various plants and several have been overexpressed in model and in crop plants, frequently leading to increased salinity tolerance (51 papers listed in Appendix S1).

Plants minimize the harmful ionic effects of Na^+^ stress by exclusion of Na^+^ from leaf tissues and by compartmentalization of Na^+^, mainly in vacuoles (Blumwald, 2000; Chanroj *et al*., 2012). *CPA1* genes can alter ionic adjustment and other regulatory processes of transgenic plants under salt stress (Chinnusamy *et al*., 2005; Jiang *et al*., 2010). However, transgenic plants have varied markedly in degree of salt tolerance and physiological change. It has been difficult to elucidate the exact mechanisms involved in *CPA1* transformation as overexpression of *CPA1s* affects many plant characteristics during normal growth as well as under salinity stress.

Phenotypes of overexpressed plants have not been assessed over consistent experimental conditions. Concentrations of NaCl and exposure time have varied greatly (Appendix S1). Gene donors and recipients have been both monocotyledonous and dicotyledonous, representing both herbaceous and woody lifestyles. Most genes used in transformation have been *AtNHX7, AtNHX1* or their orthologs in other species, but the identity of the gene within the multigenic *CPA1* family is another variable that has differed among studies.

Meta‐analysis refers to the statistical synthesis of results from multiple studies (Borenstein *et al*., 2009). The analysis allows us to characterize the consistency of a treatment effect and to estimate its magnitude much more precisely than from single studies alone. It also enables us to quantify the influence of various experimental factors on the treatment effect, factors that have been investigated across many studies and whose nuances go far beyond the capability of any one experiment. We performed a meta‐analysis on overexpression of *CPA1* genes to summarize their effects on plant responses to NaCl stress. Additionally, we examined several experimental factors (often termed moderator variables, or simply, moderators) that may have influenced the extent of the *CPA1* effect on plant response. We sought to answer the following questions: (i) What is the overall impact of *CPA1* transformation across studies on plant response to NaCl stress? (ii) Has the *CPA1* influence been more pronounced in NaCl‐stressed plants than in unstressed plants? (iii) How have particular experimental variables affected the size of *CPA1* influence? The analysis points to several potential research areas which should help scholars better understand the roles of the *CPA1* gene family in enhancing salt tolerance.

## Results

### Overall summary effects

Summary effect sizes represent the average ratio of transformed (TC) and nontransformed (NC) plants over all studies. Gene donor plants were represented by 30 species in 24 genera across the 284 studies (Appendix S1). The most highly studied *CPA1* genes were from *Arabidopsis* (109 studies). Among gene donor plant types, the most information was available for dicotyledonous plants (228 studies). Eighteen species of recipient plants were studied. The data set included eight *CPA1* gene family members with *NHX1* most examined (181 studies), followed by *SOS1* (52 studies). Single gene transformation was examined in 253 studies; the other 31 studies combined two or three genes, where one gene belonged to the *CPA1* family.

**Figure 1:**
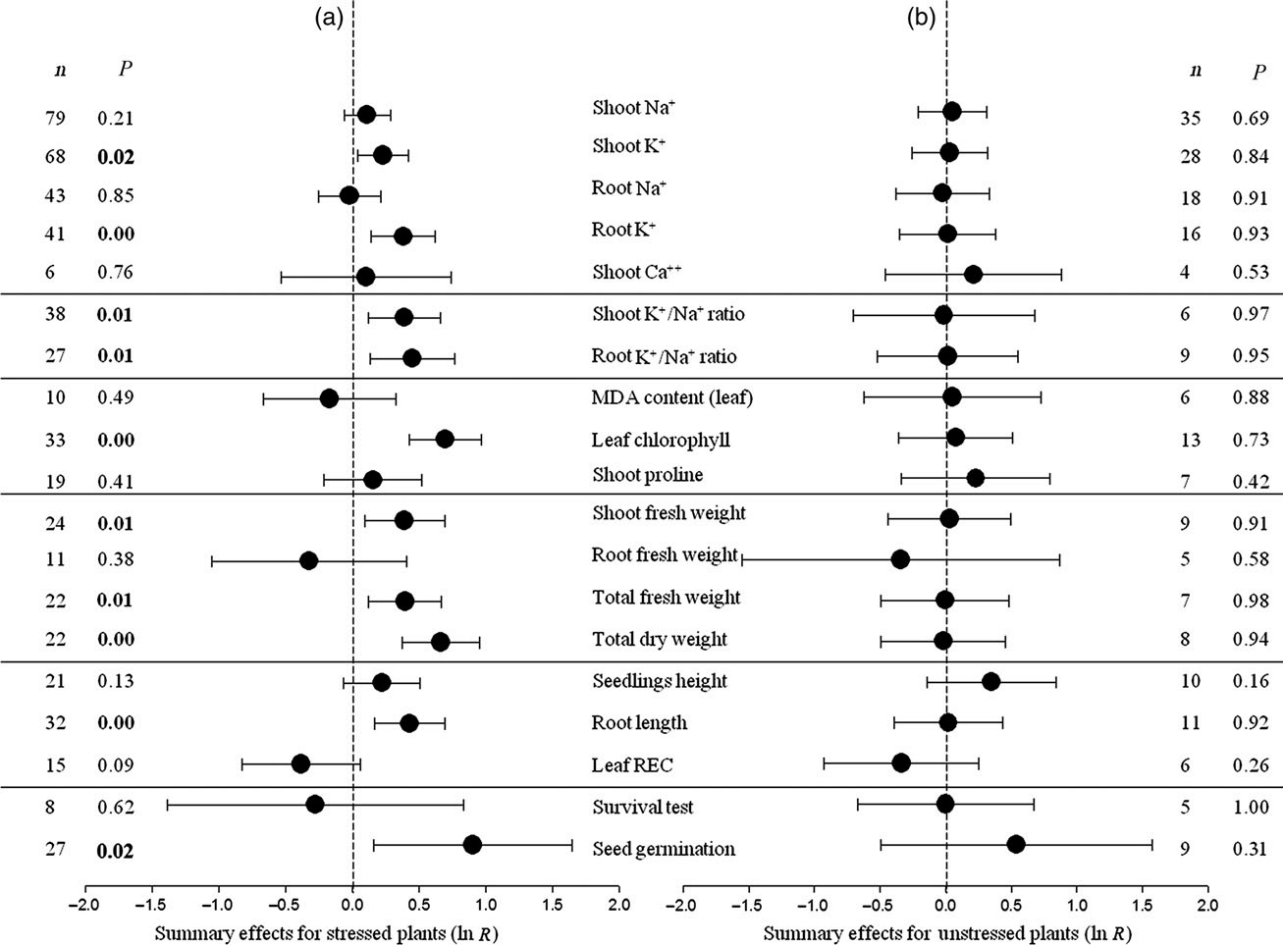
Weighted summary effect sizes (natural log of the transformed/control plant response ratios, ln *R*). Ratios are unit‐less. Horizontal bars associated with summary effects (closed circles) are 95% confidence intervals (CIs). Summary effects were analysed in plants exposed to NaCl stress (a) and unstressed plants (b). *n* is the number of studies contributing to each summary effect. *P *≤ 0.05 indicates that the summary effect was significantly different than zero (same for Figures 2, 3, 4, 5, 6, 7, 8).

**Figure 2:**
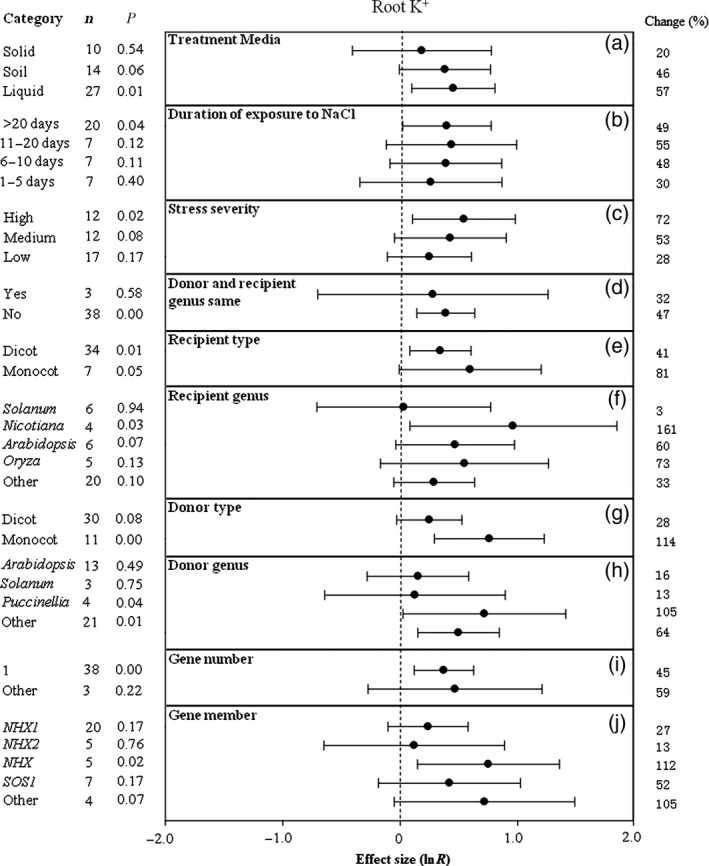
Summary effects (as natural logs, ln *R*) and 95% confidence intervals (CIs) for the influence of *CPA1* overexpression on concentration of potassium ions in roots (root K^+^). Summary effects were analysed in plants exposed to NaCl stress, with the impacts of ten moderator variables on the magnitude of the treatment effect portrayed (a–j). Category lists levels (categories or subgroups) of each moderator. Change (to the right of plots) refers to raw percentage increase in root K^+^ induced by overexpression of *CPA1*.

**Figure 3:**
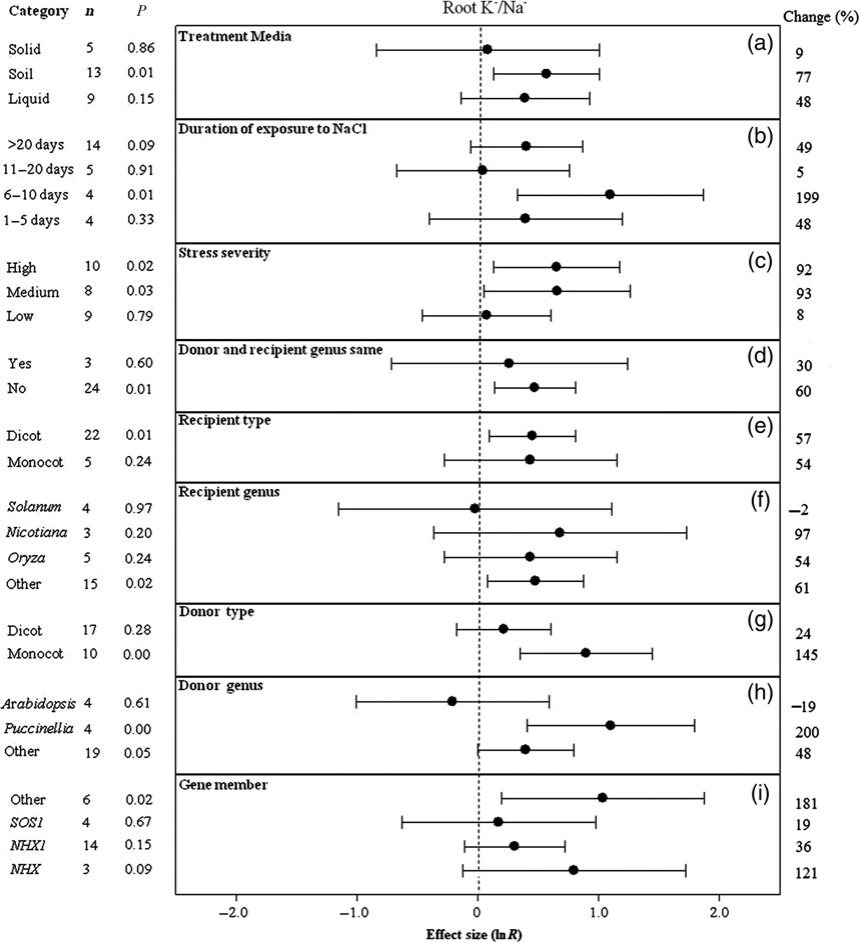
Summary effects (as natural logs, ln *R*) and 95% confidence intervals (CIs) for the influence of *CPA1* overexpression on the ratio of K^+^/Na^+^ concentrations in roots of plants exposed to NaCl. The impacts of nine moderator variables on the magnitude of the treatment effect are portrayed (a–i; gene number not analysed due to insufficient studies). Category lists levels of each moderator. Change refers to raw percentage increase in root K^+^/Na^+^ induced by overexpression of *CPA1*.

**Figure 4:**
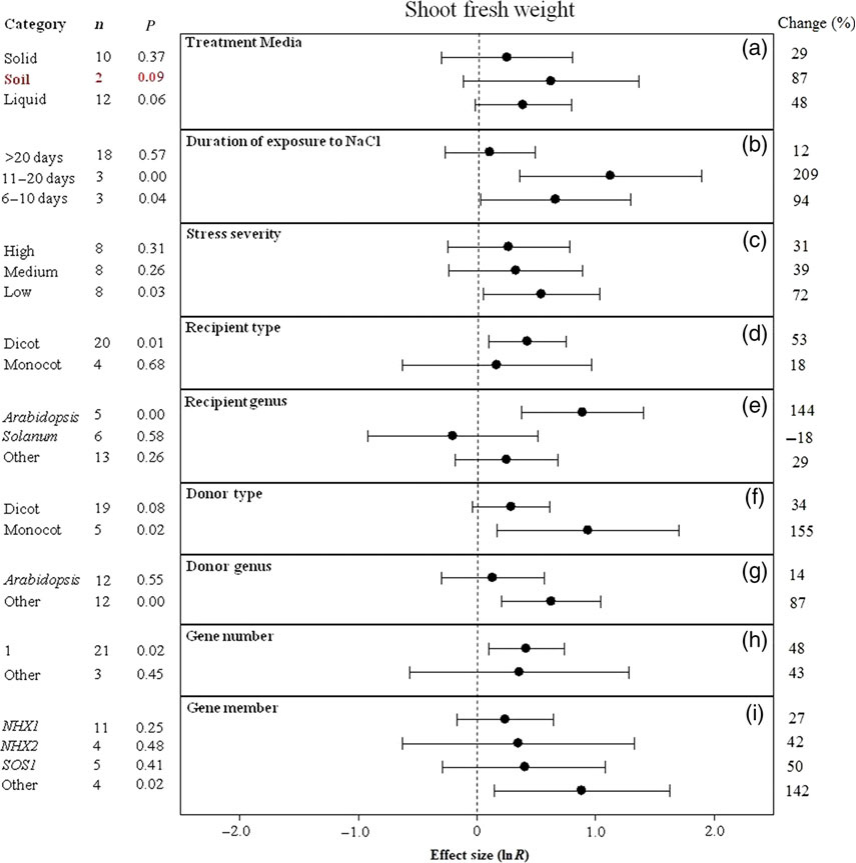
Summary effects (as natural logs, ln *R*) and 95% confidence intervals (CIs) for the influence of *CPA1* overexpression on shoot fresh weight in plants exposed to NaCl. The impacts of nine moderator variables on the magnitude of the treatment effect are portrayed (a–i; ‘same donor and recipient genus’ not analysed due to insufficient studies). Category lists levels of each moderator. Change refers to raw percentage increase in shoot fresh weight induced by overexpression of *CPA1*.

**Figure 5:**
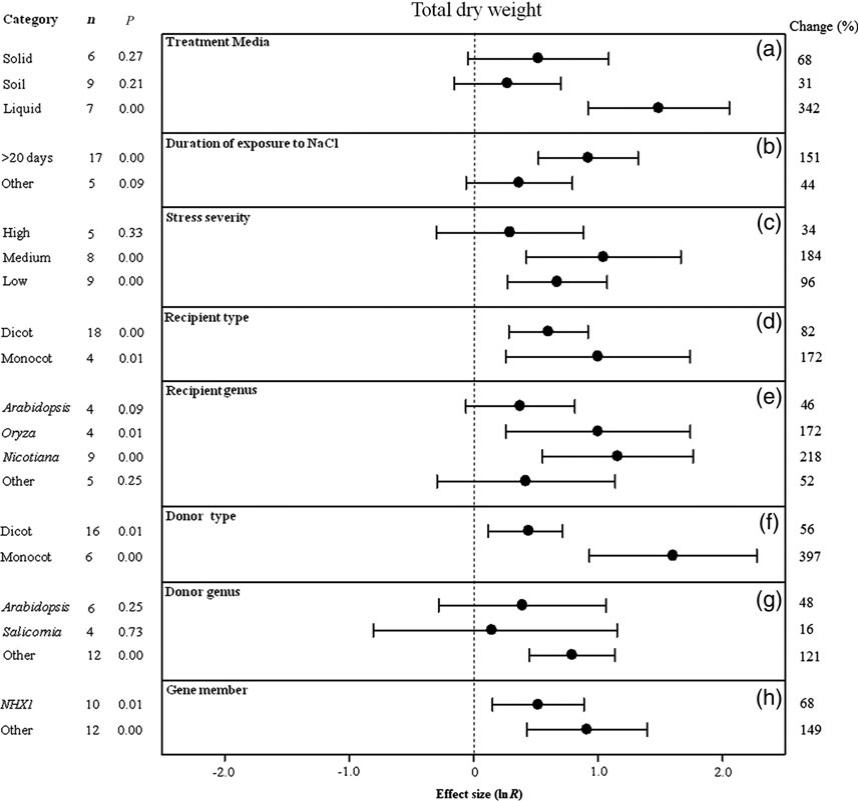
Summary effects (as natural logs, ln *R*) and 95% confidence intervals (CIs) for the influence of *CPA1* overexpression on total dry weight in plants exposed to NaCl. The impacts of eight moderator variables on the magnitude of the treatment effect are portrayed (a–h; ‘same donor and recipient genus’ and gene number not analysed due to insufficient studies). Category lists levels of each moderator. Change refers to raw percentage increase in total fresh weight induced by overexpression of *CPA1*.

**Figure 6:**
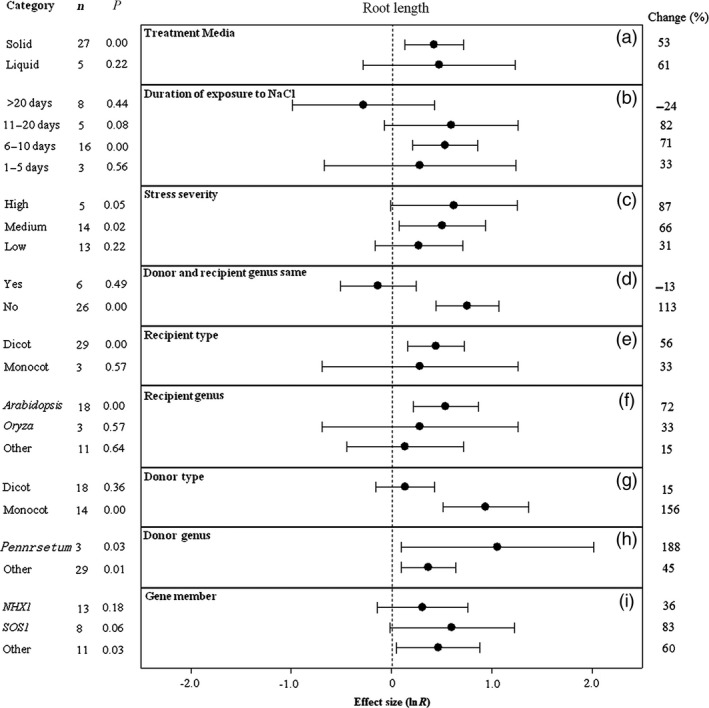
Summary effects (as natural logs, ln *R*) and 95% confidence intervals (CIs) for the influence of *CPA1* overexpression on root length in plants exposed to NaCl. The impacts of nine moderator variables on the magnitude of the treatment effect are portrayed (a–i; gene number not analysed due to insufficient studies). Category lists levels of each moderator. Change refers to raw percentage increase in root length induced by overexpression of *CPA1*.

**Figure 7:**
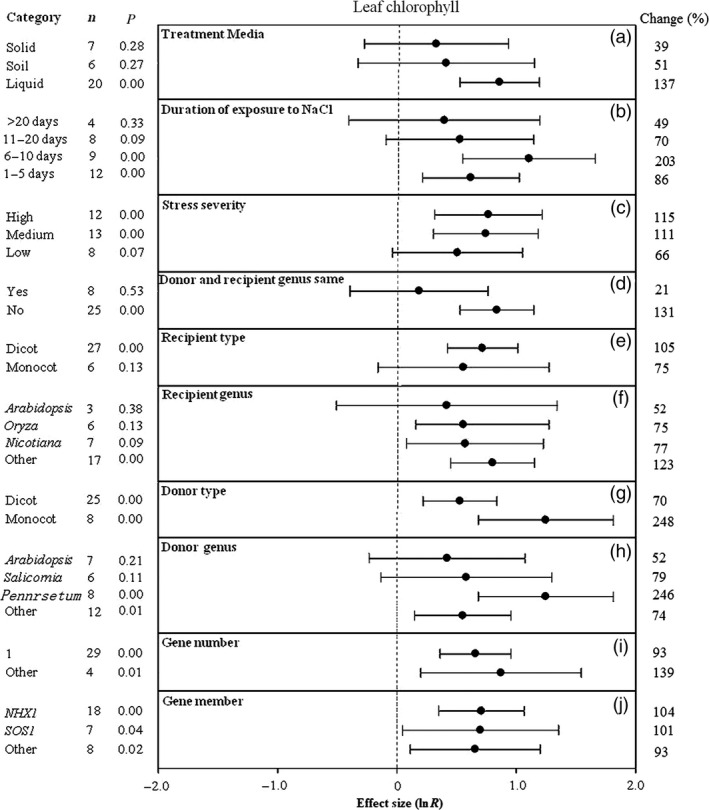
Summary effects (as natural logs, ln *R*) and 95% confidence intervals (CIs) for the influence of *CPA1* overexpression on leaf chlorophyll content in plants exposed to NaCl. The impacts of ten moderator variables on the magnitude of the treatment effect are portrayed (a–j). Category lists levels of each moderator. Change refers to raw percentage increase in leaf chlorophyll induced by overexpression of *CPA1*.

**Figure 8:**
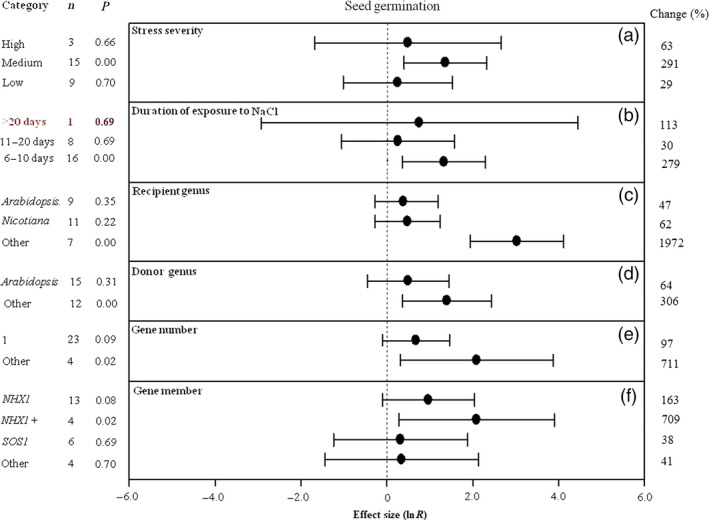
Summary effects (as natural logs, ln *R*) and 95% confidence intervals (CIs) for the influence of *CPA1* overexpression on seed germination in plants exposed to NaCl. The impacts of six moderator variables on the magnitude of the treatment effect are portrayed (a–f; some moderators not analysed due to insufficient studies). Category lists levels of each moderator. Change refers to raw percentage increase in seed germination induced by overexpression of *CPA1*.

Natural logs (ln *R*) of summary effect sizes are depicted in the plots (Figures 1, 2, 3, 4, 5, 6, 7, 8 and S1–S3). As the ratio of transformed/nontransformed treatment means (termed ‘response ratio’), the summary effect size reflects the relative magnitude of the treatment effect and its confidence intervals (CIs) indicate its precision. ‘Forest plot’ is the term used to refer to the graphical depiction of these unit‐less ratios and their CIs. When displayed as logs, positive values of the summary effect indicate promotive treatment effects and negative values inhibitive treatment effects. Raw percentage changes induced by *CPA1* transformation are listed in Table 1.

**Table 1:** Heterogeneity statistics for the 19 summary effect sizes under NaCl stress: *Qt*, total observed variation among studies; *P*_hetero_, probability that *Qt* was due entirely to sampling error and not to real variation among studies; changes to summary effects caused by foreign *CPA1* genes, transformed from ln *R* to raw percentages. Analysis was conducted on log‐transformed values (ln *R*) from each study, and raw values were provided below as percentage changes

Summary effect size	Qt	*P*_hetero_	TC‐induced change (%)
Shoot Na^+^	34.169	0.0	12
Shoot K^+^	18.685	0.0	**26**
Root Na^+^	16.556	0.0	−2
Root K^+^	11.686	0.0	**46**
Shoot Ca^2+^	0.546	0.0	11
Shoot K^+^/Na^+^ ratio	7.937	0.0	**47**
Root K^+^/Na^+^ ratio	13.300	0.0	**57**
MDA (leaf)	1.985	0.0	−16
Leaf chlorophyll	15.559	0.0	**100**
Shoot proline	8.720	0.0	17
Shoot fresh weight	10.573	0.0	**48**
Root fresh weight	7.917	0.0	−28
Total fresh weight	11.256	0.0	**48**
Total dry weight	18.627	0.0	**94**
Seedlings height	3.720	0.0	25
Root length	30.680	0.0	**53**
Leaf REC	1.934	0.0	−32
Survival test	6.833	0.0	−24
Seed germination	16.585	0.0	146

For TC‐induced changes (changes induced by *CPA1* transformation), bold text signifies change was significant (*P *≤ 0.05), with positive values indicating TC‐induced promotion and negative values TC‐induced inhibition. The magnitude and precision of each effect size listed below is illustrated in Figure 1.

In meta‐analysis, greater emphasis should be placed on the magnitude and precision of the summary effects than on tests of their statistical significance and *P* values (Cooper, 2009; Cumming, 2012). Scientific significance of the size of the treatment is of greater importance than its statistical significance, owing often to lack of adequate statistical power. However, many meta‐analysts in plant biology and ecology rely on *P* values in interpreting their analyses. We have endeavoured to combine these perspectives in summarizing our findings.

*CPA1* transformation had statistically significant impacts for 10 of the 19 plant characters examined under salt stress (Figure 1a: *P *≤ 0.05, CIs did not cross‐zero), increasing values by 25% or more (Table 1). Seed germination has been most affected by transformation, about 2.5x higher in TC than in NC plants. Chlorophyll content and total dry weight were doubled by transformation. Root K^+^/Na^+^ ratio and root length were also markedly increased in TC plants, by over 50%. Transformation had negligible impact on root Na^+^ concentration and leaf MDA content. Root fresh weight, plant survival and leaf REC were each substantively reduced in TC plants relative to their NC counterparts, by 24%–31%; small numbers of studies led to relatively large CIs and statistical insignificance for these summary effects. *CPA1* transformation has had no statistically significant impacts (*P *≥ 0.05) on these plant characteristics in the absence of NaCl (Figure 1b), likely due to imprecision (low numbers of studies) in some instances (seed germination).

### Moderator variables

Heterogeneity refers to true differences among studies in treatment outcomes (as opposed to variation due to sampling error within studies). Moderator analysis is performed to understand heterogeneity: to determine which experimental variables (moderating variables, or ‘moderators’) have had particularly large impacts on the treatment outcomes and which have had trivial or no effects. We conducted moderator analysis of the ten summary effects that were significantly affected by *CPA1* transformation (Table 2, Appendix S1). Figures 2, 3, 4, 5, 6, 7, 8 depict the influence of the moderator levels (categories) on those summary effects that have shown the most sensitivity to the moderators. None of the summary effects exhibited statistically significant heterogeneity (*P*_hetero_ > 0.10, Table 1); a *P*_hetero_ value for the *Q*‐test of <0.1 has been used to indicate significant heterogeneity in the summary effect sizes (Iacovelli *et al*., 2014). This led to values of zero in *I* ^2^ for each summary effect. *I* ^2^ is a statistic that indicates the percentage of variation due to real differences in outcomes among studies. Here, too, we note the cautions of meta analyst (Borenstein *et al*., 2009), who point out that while a significant heterogeneity *P* value or positive *I* ^2^ provides evidence that subgroups differed among trials (that there were real differences among studies), the converse does not hold. A *P* value above 0.1 does not provide evidence that moderator level studies are consistent among studies. Again, lack of significance may frequently be due to low statistical power. Even substantial real dispersion of true effects might yield *P*_hetero_ **> 0.1 with a small number of studies or large within‐study variance, often the case in plant biology meta‐analyses.

**Table 2:** Heterogeneity *P* values (*P*_hetero_): probability that moderators (experimental variables) accounted for real differences in treatment effects among studies

Moderator	Effect size									
	Shoot K^+^	Root K^+^/Na^+^	Root K^+^	Shoot K^+^/Na^+^	Leaf chlorophyll	Shoot fresh weight	Total fresh weight	Total dry weight	Root length	Seed germination
Treatment media	0.59	0.62	0.75	0.61	0.24	0.73	0.25	<0.01	0.90	–
Duration of exposure to NaCl	0.71	0.26	0.98	0.83	0.38	0.04	0.39	0.06	0.21	0.61
Stress severity	0.37	0.23	0.58	0.83	0.74	0.72	0.27	0.23	0.61	0.36
Recipient type	0.78	0.97	0.45	0.37	0.69	0.56	–	0.33	0.76	–
Recipient genus	0.85	0.83	0.54	0.64	0.81	0.04	0.30	0.14	0.47	<0.001
Donor type	0.20	0.05	0.07	0.10	0.03	0.13	–	<0.01	<0.01	–
Donor genus	0.76	0.05	0.42	0.15	0.18	0.18	0.26	0.35	0.17	0.21
Donor and recipient genus same	0.88	0.69	0.83	0.78	0.05	–	–	–	<0.001	–
Gene number	0.91	–	0.81	–	0.57	0.95	–	–	–	0.16
Gene member	0.50	0.34	0.51	0.79	0.99	0.52	0.99	0.21	0.74	–

*P*_hetero_ **≤ 0.1 signifies that observed variation is not due solely to sampling error (expected variation). Dashed entries indicate that insufficient data were available to perform an analysis. Each moderator was examined in relation to the ten summary effect sizes (ratios of treatment/control plants) significantly impacted by *CPA1* overexpression. Appendix S1 provides associated *Qm* values (between‐study variation); *n* (sample size); *df* (degrees of freedom, levels within a moderator).

### Cations

The influence of several moderators on the extent to which *CPA1* transformation affected root K^+^ in salt‐stressed plants is illustrated in Figure 2. Greater root K^+^ has been observed when experiments used liquid media than when plants were grown on soil or solid media (Figure 2a). It has generally taken a few days for root K^+^ to increase and then plateau after exposure to NaCl stress; the average TC‐induced increase over 1–5 days is 30% with the increase levelling off at 50–55% (Figure 2b). Greater stress severity has been associated with higher TC‐induced root K^+^, with low, moderate and severe stress giving average increases of 28%, 53% and 72%, respectively (Figure 2c). Monocotyledonous plants have been much more sensitive to the process than dicots; root K^+^ was twice as high in recipient monocot than dicot plants and 4x as high when gene donors were monocots than when they were dicots (Figure 2e, g). Taxonomy has also mattered; recipient and donor genera have had marked effects on the outcome of transformation. Root K^+^ has shown the greatest response in transformed *Nicotiana*, and response has been largest when genes came from *Puccinellia* (Figure 2f, h). *NHX* has had several‐fold greater impact than *NHX1* or *NHX2* and about twice the impact of *SOS1* (Figure 2j). Study numbers are low for most of these comparisons and so summary effects are imprecise (large CIs) and in most cases not statistically significant.

Unlike root K^+^, K^+^/Na^+^ in roots of transformed plants has been higher when experiments were conducted in soil than on solid or liquid laboratory media (Figure 3a). Also unlike root K^+^, root K^+^/Na^+^ has peaked dramatically in TC plants after 6–10 days of stress then subsided (Figure 3b). The impact of *CPA1* transformation on root K^+^/Na^+^ has been over 10× greater at medium and high stress than at low stress (Figure 3c). When gene donors and recipients have been of different genera, the impact of transformation has been about twice as large as when donor and recipient were of the same genus (Figure 3d). Transformed monocot and dicot recipient plants have had similar root K^+^/Na^+^, but this cation ratio has been increased substantially when donors were monocots (Figure 3e, g). As for root K^+^, *Nicotiana* and *Puccinella* have also been the most responsive genera to transformation for root K^+^/Na^+^ increases (Figure 2f, h) and *NXH* the most influential *CPA1* gene member (Figure 3i).

### Size characteristics

The effect of transformation on increasing shoot fresh weight and total dry weight of salt‐stressed plants has not been consistent across treatment media, with shoot fresh weight higher in soils (only two studies) and total dry weight considerably higher in liquid media (Figures 4a, 5a). Shoot fresh weight has been at least twice as large at moderate stress durations (11–20 days) than at shorter or longer durations (Figure 4b). Averaged across studies, both shoot dry weight and total dry weight have been almost 3× larger at low stress severity than at the most severe stress, with variable response at medium severity (Figures 4c, 5c). Monocot recipients exhibited lower shoot fresh weight but higher total dry weight with *CPA1* overexpression (Figures 4d, 5d). Biomass of salt‐stressed plants has been much more affected by transformation when donors were monocots (Figures 4f, 5f). The TC‐induced growth increases differed among recipient and donor genera but inconsistently (Figures 4e, g, 5e, g). Transformation effects on biomass have been mostly similar among the *NHX* and *SOS1* genes (Figures 4i, 5h). Across moderators, study numbers were small for these biomass effect sizes, resulting in large confidence intervals and low precision. There was much overlap among levels of most moderators; more studies are needed to resolve moderator influences.

Media type has not affected the magnitude of the TC‐induced increase in root length (Figure 6a). Intermediate exposure times have caused transformation to increase root length much more than the shortest or longest durations (Figure 6b). Also as for most effect sizes portrayed in earlier figures, greater stress severity has increased the TC‐induced impact, with the increases associated with high severity averaging about 3x greater than those for low severity (Figure 6c). *CPA1* transformation has more than doubled root length when gene donor and recipient plants subjected to NaCl stress have been of different genera (Figure 6d). When donors and recipients were of the same genus, transformation has had a slightly negative effect. Recipient dicots and monocots have not differed in TC‐induced impact on root length (Figure 6e), while monocots have clearly had much more impact than dicots as gene donors (Figure 6g). Root length of recipient *Arabidopsis* appears to have been somewhat more responsive than for other genera (Figure 6f). *Pennisetum* appears to be a promising *CPA1* donor genus, imparting a dramatic TC‐induced 188% increase in root length to recipient plants (Figure 6h). Among *CPA1* gene family members, *SOS1* has tended to have the most influence on root length of salt‐stressed plants (Figure 6i).

### Other characteristics

The outcomes of transformation on leaf chlorophyll content have been most evident in association with liquid media, with the TC‐induced impact averaging 39%, 51% and 137% for solid, soil and liquid media, respectively (Figure 7a). Again, the most dramatic impact has occurred at intermediate durations. Transformed plants exposed to NaCl stress for 6–10 days showed threefold (200%) increases in leaf chlorophyll, as opposed to more modest increases at longer time periods and at 1–5 days (Figure 7b). TC‐induced stimulation of leaf chlorophyll at the lowest stress severity was about half that at the two higher levels of severity (Figure 7c). As in root length and other effect sizes, transformation has had much greater effect on leaf chlorophyll when donor and recipient genera have differed relative to experiments in which donor and recipient were of the same genus (Figure 7d). Dicot and monocot recipient plants have not differed greatly in their TC‐induced chlorophyll response (Figure 7e), but monocots have tended to be much more influential as donors (Figure 7g). Again, *Arabidopsis* has tended to be among the least responsive species to *CPA1* transformation (Figure 7f, h). Multiple gene numbers have tended to impart more salt tolerance than single gene transformation (Figure 7i). *NHX1*,* SOS1* and other *CPA1* genes have affected leaf chlorophyll similarly, doubling it relative to controls (Figure 7j).

Seed germination data have been reported less frequently than other effect sizes, allowing less moderator analysis as well as resulting in large CIs. NaCl stress of medium severity has resulted in much greater TC‐induced impact on seed germination than high and low severity (Figure 8a). Again, a much greater impact of *CPA1* transformation has occurred at the moderate stress exposure (6–10 days) compared to longer duration, 11–20 days and >20 days (Figure 8b). Donor and recipient genera other than *Arabidopsis* have been much more affected by the *CPA1* genes (Figure 8c, d); other recipient genera combined showed a phenomenal TC‐induced increase in seed germination of 21‐fold (1972%). Transfer of multiple genes has resulted in about 8× (711%) greater impact than single gene transfers (97%) (Figure 8e), with the *NHX1* gene again having substantially greater effect than *SOS1* and other genes (Figure 8f).

### Publication bias

Publication bias is the term applied to research in the published literature that is systematically unrepresentative of all completed studies (Rothstein *et al*., 2005). A concern about bias stems from the possibility that significant treatment differences may be more likely to be published than nonsignificant findings. Several statistical methods developed to test for potential bias involve exploring the relationship between study effect size and precision. The idea is that studies with smaller sample sizes (and hence higher variance) will tend to have larger effect sizes than larger studies with greater precision; greater treatment differences are required for statistical significance as sample size decreases.

We found little evidence of publication bias in the parameters commonly used to test for it, for any of the summary effects. Funnel plots indicated that studies displayed the expected dispersion around the overall summary effect. Within the Begg and Mazumbar rank correlation test, all summary effects except shoot Ca^2+^ had absolute Kendall tau values below 0.3, indicating little concern for bias (no tendency for effect sizes to increase as study size decreases). The Duvall and Tweedie trim and fill analysis indicated no concern that publication bias has resulted in an inflated summary effect. The purpose of the fail‐safe calculation is to estimate whether publication biases (if they exist) can be safely ignored (Rosenberg, 2005). In all cases, the Orwin's failsafe N was much higher than the classic Rosenthal threshold number of 5*k *+ 10. In other words, a very large (and very unlikely) number of missing studies would be needed to reduce the treatment effect to <4%, for any of the summary effects. The Egger's *P* values did suggest the possibility of bias for shoot Na^+^, shoot Ca^2+^, shoot fresh weight and total fresh weight. This one test should be viewed within the context of the several other measures that do not indicate a concern for bias. Additional details and tests are provided in Appendix S2.

## Discussion

The involvement of *NHX1*,* SOS1* and other *CPA1* genes in plant response to NaCl stress has been well documented (An *et al*., 2007; Ma *et al*., 2015). Sodium toxicity disrupts ion homoeostasis and consequently cellular metabolism (Ahmad and Maathuis, 2014). Many cytosolic enzymes that are activated by K^+^ are inhibited by Na^+^ (Flowers *et al*., 1977), and high cytosolic K^+^/Na^+^ ratio helps sustain normal metabolism. Na^+^/H^+^ antiporters move sodium ions across membranes by exchanging H^+^ for Na^+^ (Adem *et al*., 2015). Genes in the *CPA1* family encode Na^+^/H^+^ exchangers that act in compartmentalizing Na^+^ and thereby reducing its accumulation in the cytosol (Apse *et al*., 1999; Serrano and Rodriguez‐Navarro, 2001). Maintaining high K^+^ is important in sodium tolerance, and *NHX* genes have been found to assist K^+^ homoeostasis (Barragán *et al*., 2012). *CPA1* gene members also act in control of long‐distance transport of Na^+^ (Shi *et al*., 2002), by retrieving sodium from root xylem and sequestering it in root vacuoles (Martínez‐Alcántara *et al*., 2015) and potentially by exporting it back to the soil solution (Nie *et al*., 2015). Favourable ion regulation protects cells in several ways, for example by enhancing proline accumulation, preserving membrane integrity and increasing the scavenging of reactive oxygen species (Fan *et al*., 2015). The value of the meta‐analysis, beyond the confirming the overall promotive influence of ectopic *CPA1* overexpression on salt tolerance, gives the average magnitude of the influence across studies and enables us to see which plant attributes have been most influenced by overexpression. Perhaps more importantly, the analysis gives perspective on which experimental circumstances and plant taxa have been associated with the greatest impacts of overexpression and which with minimal impact.

The positive TC‐induced effects on phenology have occurred at all durations but have been greatest for several plant characteristics when measured with exposures of intermediate duration. The *CPA1* impact is usually substantial in the first days of exposure, becomes highest at 6–20 days then subsides, often becoming negligible after about three weeks. This suggests that overexpression of *CPA1* genes may provide only moderate salt tolerance because the amount of Na^+^ that can be moved into vacuoles has limits; ultimately, compartmentation is swamped. When Na^+^ accumulation reaches the limit of vacuolar capacity, likely the case under severe salinity or long‐term exposure to moderate salinity, the impact of *CPA1* overexpression subsides. This may explain why no highly salt‐resistant crop varieties have been developed by overexpression of *CPA1* genes. Still, there are many instances in which modest boosts in resilience may prove very worthwhile. For example, achieving maximum protection at 2–3 weeks may have particular advantage for transplants, which are especially vulnerable during the first weeks after transplantation to the field or into containers.

Adem *et al*. (2015) suggested that several genes need to be pyramided for antiporter transformation to achieve a meaningful effect. The meta‐analytic perspective across the literature provides some support for this idea, in that the TC‐induced improvement was largest for some plant characteristics when more than one gene was transferred to recipient plants relative to transformation with a single gene. Low study numbers for these comparisons resulted in imprecise summaries (large CIs), but three of the four characteristics examined root K^+^, leaf chlorophyll and seed germination clearly displayed this trend. The functions of *CPA1* genes for plant salt tolerance can be classified into two main categories based on the locations of proteins and the different pathways in the molecular evolution of stress tolerance (Bassil and Blumwald, 2014; Pires *et al*., 2013). Therefore, it seems reasonable that transferring two types of genes into one recipient plant may potentially have an additive effect in reducing Na^+^ toxicity. This should be a fruitful area for additional research.

Why has *CPA1* transformation failed to improve stress tolerance in some studies? Adem *et al*. (2015) pointed to several possibilities for why the phenology or behaviour of transformed plants may not differ from controls: low activity of vacuolar enzymes resulting in an insufficient proton gradient; inability of transgenic plants to prevent a passive leak of sodium from vacuoles back into cytosol; insufficient ATP to support proton pumping; and improper folding or incorrect targeting of antiporter proteins. It is possible that one or more of these processes have been associated with those moderator subgroups that have shown relatively low TC‐induced impact. This is another avenue that warrants future research.

Other promising investigation involves plant taxa. The identity of the donor plant has influenced the efficacy of *CPA1* transformation on each effect size. The Na^+^/H^+^ antiporter gene in *A. thaliana* was the first *NHX* homolog to be cloned (Gaxiola *et al*., 1999), and much subsequent overexpression work has used *Arabidopsis* as a model plant, providing pioneering breakthroughs (Khan *et al*., 2015). Yet the meta‐analysis shows that *Arabidopsis* is among the least effective donor genera and only slightly more efficacious when it has been the transgenic recipient. The inconstancy of impact of *Arabidopsis* in *CPA1* overexpression studies raises the question of whether it is the best model species for evaluating *CPA1* gene function in other species. The reliability of endogenous versus ectopic overexpression also warrants more investigation.

The association of *Arabidopsis* with lower effectiveness of *CPA1* transformation may at least partially relate to plant type. *Arabidopsis*, a dicotyledonous plant, is the most frequently studied donor genus. For each of the six plant characteristics for which plant types were compared, transformation had a markedly greater effect when the donor plant was monocotyledonous than when it was dicotyledonous. That this was not the case for recipient plants is likely fortunate for agriculture. Both dicot and monocot recipient plants have benefited by transformation, maintaining an open field for improvement of salt tolerance in both crop types.

The meta‐analysis indicates that the transgenic effect on K^+^ has generally been more pronounced than on Na^+^; *CPA1* overexpression has increased shoot and root K^+^ concentrations while maintaining shoot and root Na^+^ concentrations. The TC‐induced increase in shoot and root K^+^/Na^+^ ratio, a well‐known finding (Garciadeblás *et al*., 2007; Qi and Spalding, 2004), has been statistically significant and dramatic when viewed across the literature. Calcium has been less studied. *SOS1* is the most crucial gene in the *SOS* pathway associated with plant salt‐induced responses, and Ca^2+^ has been identified as the key signal in the pathway (Ditta, 2013). However, less than ten studies on *SOS1*‐transformed plants included measurements of Ca^2+^ concentration in plant tissues. Previous physiological studies have shown that the *SOS1* gene is related to Na^+^ exclusion, especially in the root tissue (Bassil and Blumwald, 2014; Bassil *et al*., 2012; Jannesar *et al*., 2014; ), but Ca^2+^ concentration in the root has rarely been measured in these *CPA1*‐overexpressed plants. The *CPA1* gene family belongs to the *CPA* super‐family (Brett *et al*., 2005; Mäser *et al*., 2001); therefore, overexpression of these genes should potentially influence many ions in addition to Na^+^ and K^+^, such as Ca^2+^ and Mg^2+^ (Kurusu *et al*., 2013; Pandey *et al*., 2013). Only two of 51 studies of root behaviour (Bao *et al*., 2014; Yadav *et al*., 2012) provided root Ca^2+^ measurements (which did not meet our effect size inclusion criterion of a minimum of ten studies). Mg^2+^, another bivalent ion, plays an important role in chlorophyll function (Walker and Weinstein, 1994). The meta‐analysis confirmed that *CPA1* transformation has led to dramatically higher chlorophyll concentration when plants were exposed to salinity. Few studies have reported Mg^2+^ concentrations, and it is unknown whether the impact on chlorophyll relates to maintained Mg^2+^ concentration or to other factors. The measurement of bivalent ion changes in future studies can clarify the contribution of these ions to salt tolerance in relation to overexpression of *CPA1* genes.

Among transgenic strategies examined thus far, Na^+^/H^+^ antiporters offer the best mechanism for ion homoeostasis in plants subjected to NaCl stress (Khan *et al*., 2015; Rozema and Schat, 2013). The weighted average view across the literature confirms that using *CPA1* overexpression to fortify crop plants against stress is a promising biotechnology, especially as its impact has tended to increase as salt stress severity increases. The analysis points to several areas that warrant further inquiry, in addition to those noted above. Seed germination may represent an especially profitable area for investigation, as it has been the attribute most affected by overexpression, not only in salt‐stressed plants but in unstressed plants. *CPA1* overexpression has had its greatest impact during intermediate durations, roughly coinciding with germination times. Across the 27 studies which measured seed germination, all of the genes were isolated from dicotyledonous plants, and most of the recipient plants were also dicotyledonous. Given that monocots have tended to be more responsive to *CPA1* overexpression than dicots, it will be beneficial to learn whether germination under salt stress is impacted even more in monocot seeds. Salt can cause several kinds of physiological stress, and quite a few parameters have been evaluated in traditional physiological studies, for example antioxidant enzymes, MDA, REC, and proline and other compatible solutes (Song *et al*., 2014; Wang *et al*., 2011). However, many of these parameters have not been examined in *CPA1* overexpression studies, so it is unclear whether overexpression of a *CPA1* gene would affect these parameters; this may also be an area of promising research. Another important future research area is cropping situations. As Khan *et al*. (2015) noted, more field studies are needed to determine the extent to which laboratory findings will translate to increased yield potential in actual cropping situations.

## Materials and methods

### Data collection

Data were located through a systematic search of 12 electronic databases using Endnote and ISI Web of Science (Thompson Reuters; additional search details provided in Appendix S1). The search was conducted on 3 October 2014 for articles dated through September 2014 using the search terms (‘*CPA1* gene family’ or ‘*NHX* gene*’ or ‘*SOS1* gene*’) AND (‘overexpress*’). The search returned 612 unique articles, all from refereed journals. Upon examination, 573 of the articles were excluded because they did not meet our inclusion criteria: data were unrelated to plants (81); *CPA1* data were not reported (329); *CPA1* genes were not overexpressed (99); they were review articles (60); either treatment or control mean was not reported (1); same data had been reported in another article (1); or the plant parameters in which we were interested were not reported (1). Thirty‐nine articles from the Web of Science search met our screening criteria. We located an additional 12 articles by examining article reference lists. In all, 51 articles were included in the meta‐analysis, written in English and Chinese and spanning 15 years (citations provided in Appendix S1). Treatment and control means with sample sizes were obtained for each study. Where sample size was not reported, we defined it conservatively as *n* = 1 if no mean statistics were provided (one study) and *n* = 2 if mean statistics were reported (12 studies). We used the final time point for studies that included data for multiple time points. Data from graphs were extracted using GetData Graph Digitizer (http://getdata-graph-digitizer.com).

Multiple treatments from one article were treated as independent studies and represented individual units in the meta‐analysis. For example, An *et al*.(2007) examined the behaviour of transformed and nontransformed plants subjected to four different NaCl concentrations, which resulted in four studies. Wang *et al*. (2007) included five different concentration NaCl (0, 150, 175, 200 and 225 mm), which resulted in five studies. Although including more than one study from an article has the disadvantage of potentially increasing the statistical dependence among studies, which for the purposes of meta‐analysis are assumed to be independent (Gurevitch and Hedges, 1999), the larger number of studies maximizes the analysis’ statistical power (Lajeunesse and Forbes, 2003). This approach has been used commonly in plant biology meta‐analyses (Klümper and Qaim, 2014; Wujeska *et al*., 2013), especially when studies address the moderators being evaluated (Lehmann and Rillig, 2015).

### Effect sizes and moderators

We analysed several response variables commonly investigated in plant NaCl stress research (Figure 1). Each was incorporated into the analysis as the ratio of transgenic to nontransgenic (control) plant means. This response ratio is referred to as the effect size, and its natural logarithm, ln *R*, is used in the meta‐analysis (Borenstein *et al*., 2009):

$$\ln\;R=\ln Y_{\mathrm{TC}}/Y_{\mathrm{NC}}$$

where *Y*_TC_ and *Y*_NC_ are the means of plants that were transformed (TC) and nontransformed (NC) with a *CPA1* gene (including transformed with an empty vector), respectively. Response ratios are commonly used in meta‐analyses of plant behaviours (Schaeffer *et al*., 2013; Worchel *et al*., 2013), as they provide a standardized, unit‐less expression of treatment‐induced change. The log transformation properly balances positive and negative treatment effects across response ratios (maintains symmetry in the analysis, Borenstein *et al*., 2009). Ln *R* values above 0 indicate a TC‐induced increase in the parameter, values below 0 indicate a TC‐induced decrease in the parameter, and a value of 0 signifies a lack of effect of *CPA1* overexpression. An overall, summary effect size was computed for each of the 19 plant characteristics as the weighted mean of effect sizes from the primary studies. Chlorophyll concentration refers to leaves except for three studies in which shoot chlorophyll was reported. K^+^/Na^+^ ratios were computed when papers reported respective treatment means for K^+^ and Na^+^ concentration and did not directly provide the K^+^/Na^+^ ratio. Root and shoot data were analysed for those plant characteristics for which these were commonly reported for both organs. Generally, only leaf/shoot data were reported for relative electric conductivity (REC) and concentrations of malondialdehyde, proline and Ca^2+^.

In addition to effect size data, we collected information from each study on ten experimental variables or ‘moderators’ that may modify response to salinity stress. Moderators were of three varieties: (i) experimental conditions: treatment medium, stress severity and duration; (ii) experimental materials: type and genus of gene donor and recipient, whether donor and recipient were the same genus; and (iii) character of foreign genes: identity of the *CPA1* genes, number of genes transferred. Moderators were examined to determine whether the effect of *CPA1* overexpression has been more pronounced in some experimental contexts than in others. Each moderator contained at least two levels (categories), and a level of a moderator was included in the analysis if data were available for it from at least three studies from more than one article. Categorical levels not meeting these criteria were grouped into a level analysed as ‘other’ and included in the analyses if ‘other’ contained at least three studies from more than one article.

When an experiment included three or more levels of a NaCl stress treatment, we scored severity of the stress as ‘low’, ‘medium’ and ‘high’, in an effort to evaluate whether the summary effects were impacted differently by different degrees of stress. For example, if an experiment employed four levels of NaCl stress, 100, 200, 300 and 400 mm NaCl, we defined the 100 mm treatment as low, 400 mm treatment as high and 200 and 300 mm treatments as medium.

### Meta‐analysis

Meta‐analyses were performed using Comprehensive Meta‐Analysis (CMA) software (Version 3.0, Biostat, Englewood, NJ; 2014). Individual studies were weighted using nonparametric variance:

$$V\;\ln\;R=\left(n_{\mathrm{TC}}+n_{\mathrm{NC}}\right)/\left(n_{\mathrm{TC}}*n_{\mathrm{NC}}\right)$$

where *V* ln *R* is the variance of the natural log of the response ratio *R* and *n*_TC_ and *n*_NC_ are the sample sizes of the TC and NC treatments. Standard errors or standard deviations were not reported for most studies nor was sufficient information given in many instances to estimate these from ANOVA mean separation test values. It is not uncommon for measures of dispersion to have been omitted from publications in plant biology, which makes calculating weight based solely on sample size (nonparametric variance) a necessity. Treatment summary effects were considered significant if their 95% confidence intervals did overlap zero. Presence or absence of heterogeneity (real or true variation in effects) was assessed with the *Q* statistic (a measure of weighted squared deviations) and estimated using *I* ^2^ (a descriptive index that estimates the ratio of true heterogeneity to total variation across the observed effect sizes) (Borenstein *et al*., 2009). Potential publication bias was assessed statistically with Begg and Mazumbar rank (Kendall) correlation and Egger's linear regression (Begg and Mazumdar, 1994; Sterne and Egger, 2005). It was represented graphically with funnel plots of effect sizes versus their standard errors (Borenstein *et al*., 2009). A fail‐safe method was used to ask whether the entire summary effect may be attributed to bias. We employed the Orwin's fail‐safe N approach (Borenstein, 2005), considered an improvement on the original Rosenthal fail‐safe N method (Rosenthal, 1979). The Duval and Tweedie iterative trim and fill method was used to demonstrate how the summary effect size would shift if apparent bias were to be removed (Duval and Tweedie, 2000). Additional details about publication bias tests are provided in Appendix S1.

## Supporting information


**Figure S1** Summary effects (as natural logs, ln *R*) and 95% confidence intervals (CIs) for the influence of *CPA1* overexpression on shoot K^+^ concentration of plants exposed to NaCl.

**Figure S2** Summary effects (as natural logs, ln *R*) and 95% confidence intervals (CIs) for the influence of *CPA1* overexpression on shoot K^+^/Na^+^ ratio of plants exposed to NaCl.

**Figure S3** Summary effects (as natural logs, ln *R*) and 95% confidence intervals (CIs) for the influence of *CPA1* overexpression on shoot K^+^/Na^+^ ratio of plants exposed to NaCl.

**Appendix S1** Details on the CPA1‐overexpression studies used in the meta‐analyses, including each of the moderators used for categorical analyses, the transgenic and untransformed control means, sample sizes (n) and the value of the natural log (ln) of the response ratio with corresponding non‐parametric variances.

**Appendix S2** Analyses of publication bias.
**Table S1** Measures used in characterizing publication bias for each effect size.
