# Supplementary figures and images for: Increased salt tolerance with overexpression of cation/proton antiporter 1 genes: a meta‐analysis
Source: Plant Biotechnol J. 2016 Sep 6;15(2):162–73. doi: 10.1111/pbi.12599 (PMC5258863; doi:10.1111/pbi.12599)

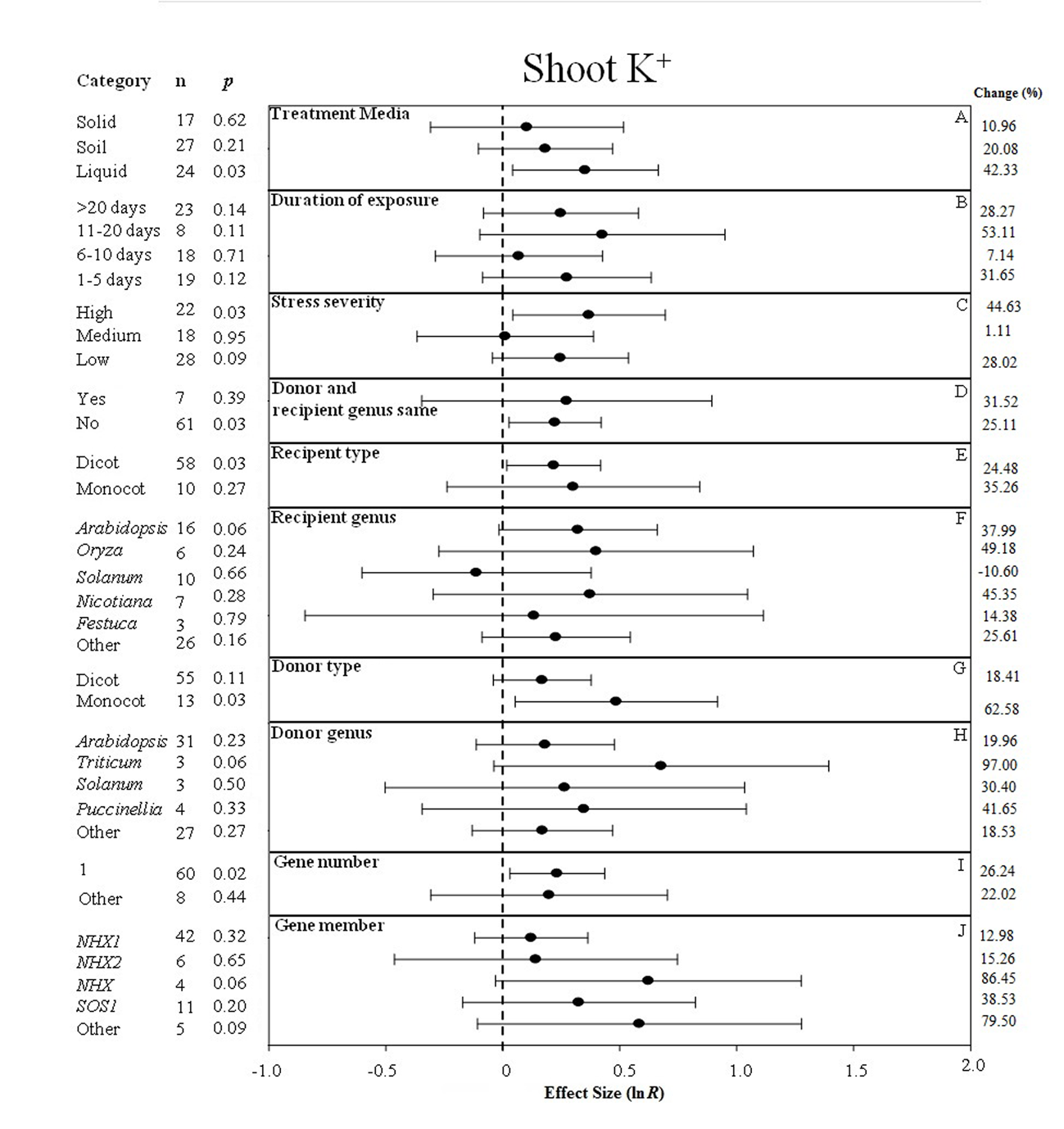

Supplement: Supplementary file 1 — Figure S1 Summary effects (as natural logs, ln R) and 95% confidence intervals (CIs) for the influence of CPA1 overexpression on shoot K+ concentration of plants exposed to NaCl. [file PBI-15-162-s004.tif]

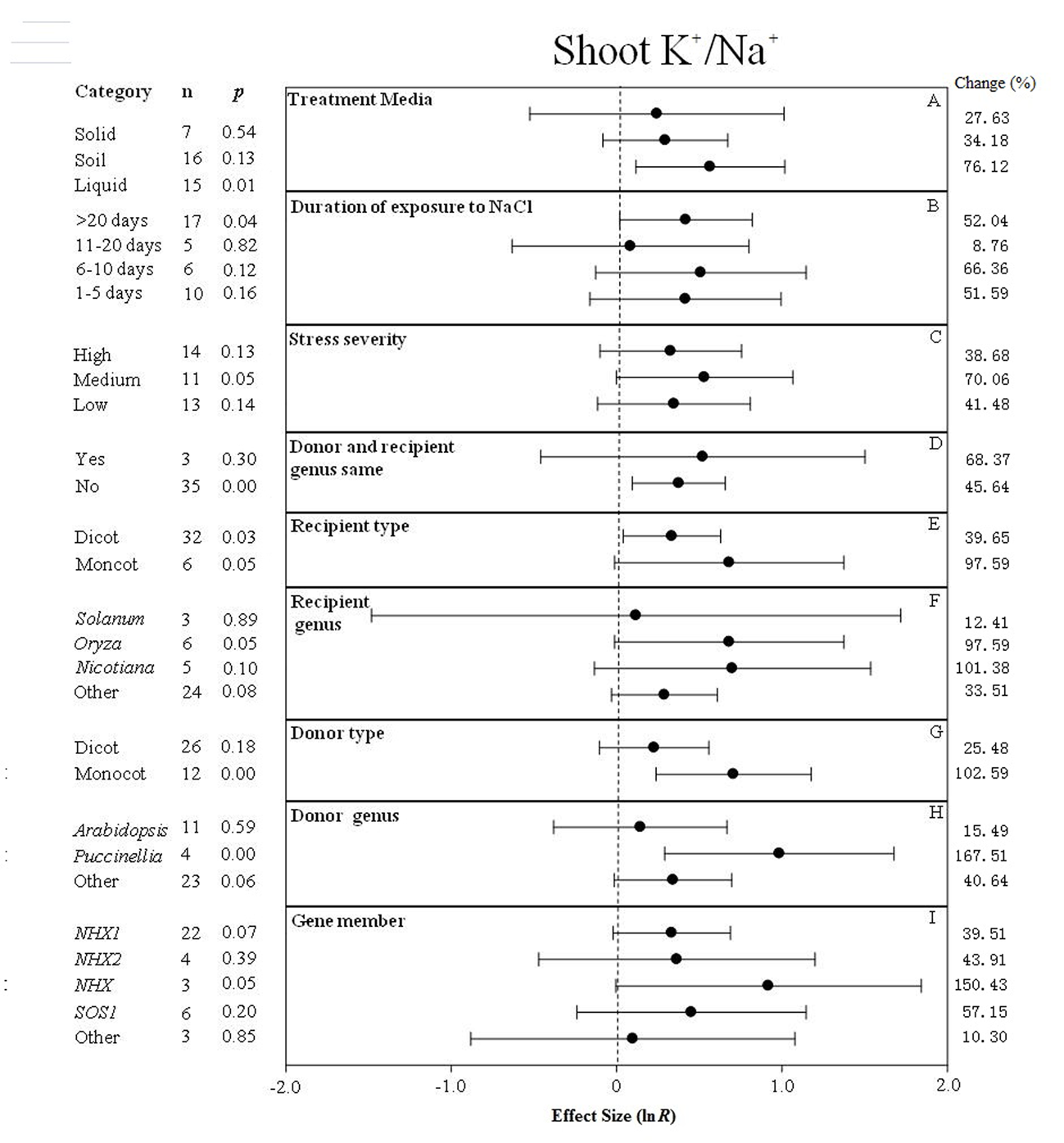

Supplement: Supplementary file 2 — Figure S2 Summary effects (as natural logs, ln R) and 95% confidence intervals (CIs) for the influence of CPA1 overexpression on shoot K+/Na+ ratio of plants exposed to NaCl. [file PBI-15-162-s005.tif]

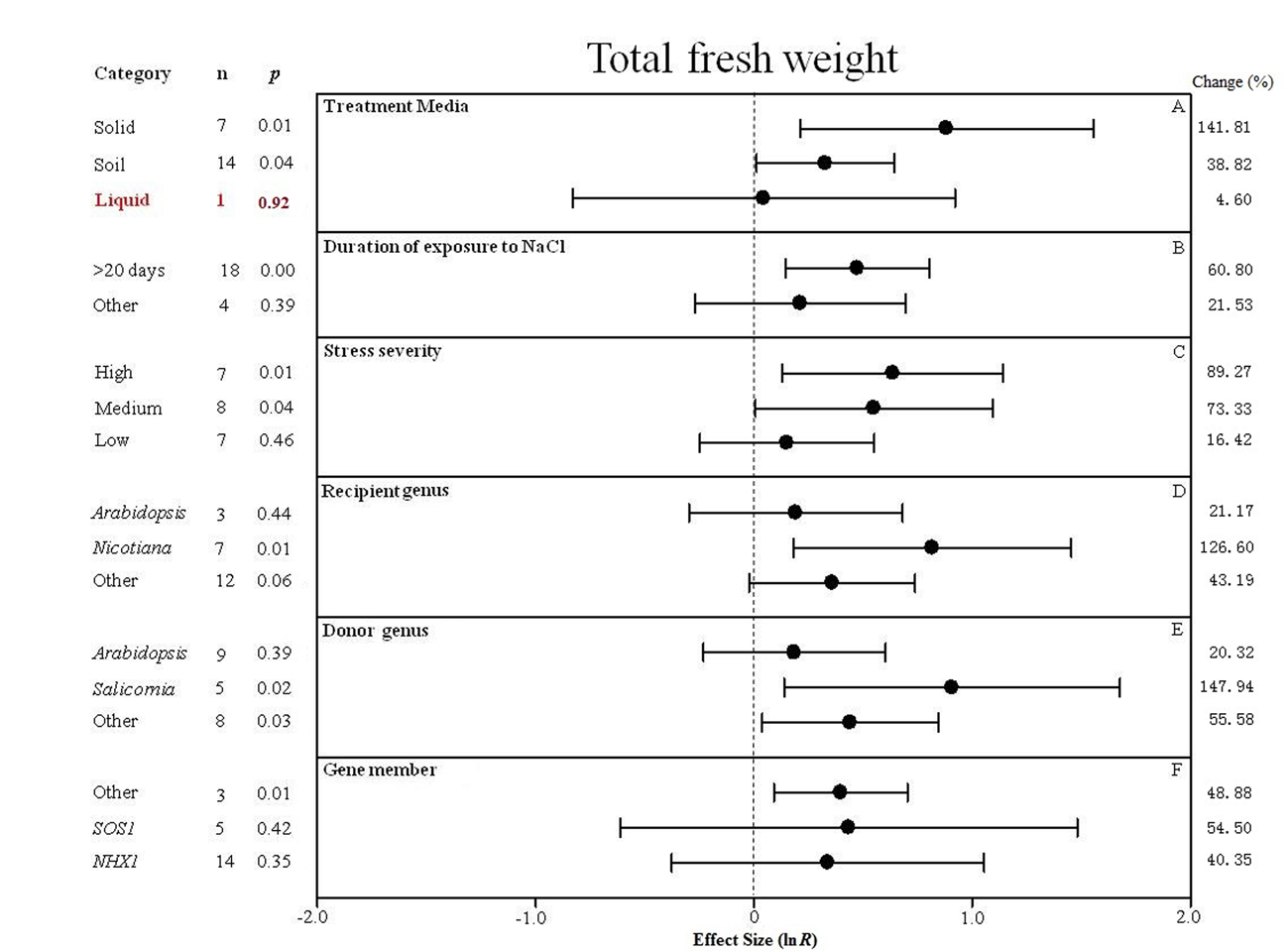

Supplement: Supplementary file 3 — Figure S3 Summary effects (as natural logs, ln R) and 95% confidence intervals (CIs) for the influence of CPA1 overexpression on shoot K+/Na+ ratio of plants exposed to NaCl. [file PBI-15-162-s003.tif]
