# Supplementary material for: Increased salt tolerance with overexpression of cation/proton antiporter 1 genes: a meta‐analysis
Source: Plant Biotechnol J. 2016 Sep 6;15(2):162–73. doi: 10.1111/pbi.12599 (PMC5258863; doi:10.1111/pbi.12599)
Supplement: Supplementary file 5 — Appendix S2 Analyses of publication bias. Table S1 Measures used in characterizing publication bias for each effect size. [file PBI-15-162-s001.doc]

**Appendix S2: Analyses of Publication Bias**

Publication bias is the term applied to research in the published literature that is systematically unrepresentative of all completed studies (Rothstein *et al.*, 2005). Literature reviews can be subject to publication bias, the standard narrative review more so than quantitative meta-analysis review (Borenstein *et al.*, 2009). But the issue is raised more often with meta-analysis, probably because this method purports to be comprehensive. The concern stems from the possibility that significant treatment differences are more likely to be published than non-significant findings. Direct evidence of publication bias is difficult to obtain yet its presence should always be checked (Sutton 2005).

Three kinds of techniques are generally used to evaluate publication bias: to detect it, to assess sensitivity of conclusions to potential bias, and to adjust conclusions for presence of potential bias (Rothstein *et al.*, 2005). We followed the recommendations of Borenstein (2005) that a thorough examination for publication bias proceed through a sequence of analyses. After examining funnel plots to get a visual sense of the data, we used rank correlation and regression procedures to test for evidence of bias or lack thereof. The Begg and Mazumdar’s test derives the rank correlation between standardized effect sizes and their standard errors (estimated from their non-parametric variances) (Begg and Mazumdar, 1994). The Egger’s linear regression method was used to assess the relation between a study’s “normal deviate” *z*, where *z* = standardized effect size/standard error, and its precision (reciprocal of variance) (Sterne and Egger, 2005). A fail-safe method was then used to ask if the entire summary effect may be attributed to bias. We employed the Orwin’s fail-safe N method (Borenstein, 2005), considered an improvement on the original Rosenthal fail-safe N method (Rosenthal, 1979). The Duval and Tweedie iterative trim and fill method was used to demonstrate how the summary effect size would shift if apparent bias were to be removed (Duval and Tweedie, 2000).

Among the methods have been developed to test for bias in a data set and adjust for it, several involve exploring the relationship between study effect size and precision. The idea is that studies with smaller sample sizes or higher variance will tend to have larger effect sizes than larger studies with greater precision. The funnel plot – individual study effect sizes as a function of their standard errors or variances – is a visual representation of this. Across summary effects in our study, most funnel plots were symmetric and give no concern for publication bias (Table S3). The Kendall tau value of 0.67 for shoot Ca++ indicates possibility of bias, as does the *p* of 0.01 for this summary effect. Kendall tau values were below 0.3 for all other summary effects with *p* > 0.05 (seedling height *p* = 0.05), indicating little concern for bias. The Egger’s linear regression method, like the Kendall rank correlation, is intended to quantify bias revealed by the funnel plot (Borenstein, 2005). The two-tailed significance tests (Egger’s *p* values) suggested the possibility of bias for shoot Na^+^, shoot Ca^++^, shoot fresh weight and total fresh weight.

A fail-safe calculation is not a method of identifying publication bias nor of accounting for bias if it exists. Its purpose is to estimate whether publication biases (if they exist) can be safely ignored (Rosenberg, 2005). Although the utility of fail-safe N methods for characterizing publication bias has been questioned (Becker, 2005), investigators in diverse fields do apply it (e.g. Freeman *et al.*, 2014; Scasta *et al.*, 2015; Sperry *et al.*, 2015). In all cases, the Orwin’s failsafe N was much higher than the classic Rosenthal threshold number of 5*k*+10. I.e., a very large number of missing studies would be needed to reduce the transformation treatment effect to less than 4%, for all summary effects.

The Duval and Tweedie trim and fill procedure imputes missing studies needed to make the funnel plot symmetrical, removing the most extreme small studies and recomputing the effect size at each iteration until the funnel plot is symmetric on either side of the new (adjusted) summary effect. To maintain proper variance, the original studies are added back into the analysis along with a mirror image for each. The adjusted value is suggestive only, as when between-study heterogeneity exists (as was the case in our meta-analysis), trim and fill may inappropriately adjust for publication bias where none exists and thereby lead to spurious changes in the summary effect (e.g. Terrin *et al.,* 2003). The main concern about missing studies is that without them, the summary effect is exaggerated. In our analysis, however, the summary value adjusted for potential missing studies is further from zero than the original value, for 16 of the 19 summary effects. Therefore, the trim and fill analysis indicates no concern that publication bias has resulted in an inflated summary effect. In fact, if there is any veracity in the suggested adjustments – if there really are missing studies – then the Duval and Tweedie analysis points to an even greater impact of transformation in improving salt tolerance.

**References**

Becker, B.J. (2005) Failsafe N or File-Drawer Number. In: *Publication bias in meta-analysis: prevention, assessment and adjustments* (Rothstein, H.R., Sutton, A.J. and Borenstein, M.), pp. 111-125. John Wiley & Sons, Ltd.

Begg, C.B. and Mazumdar, M. (1994) Operating characteristics of a bank correlation test for publication bias. *Biometrics* **50**, 1088-1101.

Borenstein M. (2005) Software for publication bias. In: *Publication bias in meta-analysis: prevention, assessment and adjustments* (Rothstein, H.R., Sutton, A.J. and Borenstein, M.), pp. 193-220. England: John Wiley & Sons, Ltd.

Borenstein, M., Hedges, L.V., Higgins, J.P.T. and Rothstein, H.R. (2009) *Introduction to meta-analysis*. Manhattan: John Wiley & Sons.

Duval, S. and Tweedie, R. (2000) A nonparametric "trim and fill" method of accounting for publication bias in meta-analysis. *J. Am. Stat. Assoc.* **95**, 89-98.

Freeman, S., Eddy, S.L., McDonough, M., Smith, M.K., Okoroafor, N., Jordt, H. and Wenderoth, M.P. (2014) Active learning increases student performance in science, engineering, and mathematics. *Proc. Nat. Aca. Sci.* **111**, 8410-8415.

Rosenberg, M.S. (2005) The file-drawer problem revisited: a general weighted method for calculating fail-safe numbers in meta-analysis. *Evolution* **59**, 464-468.

Rosenthal, R. (1979) The file drawer problem and tolerance for null results. *Psychol. Bull.* **86**, 638-641.

Rothstein, H.R., Sutton, A.J. and Borenstein, M. (2005) In: *Publication bias in meta-analysis: prevention, assessment and adjustments* (Rothstein, H.R., Sutton, A.J. and Borenstein, M.), pp. 1-8. England: John Wiley & Sons, Ltd.

Scasta, J.D., Engle, D.M., Fuhlendorf, S.D., Redfearn, D.D. and Bidwell, T.G. (2015) Meta-analysis of exotic forages as invasive plants in complex multi-functioning landscapes. *Invas. Plant Sci. Mana.* **8**, 292-306.

Sperry, S.D., Scully, I.D., Gramzow, R.H. and Jorgensen, R.S. (2015) Sleep duration and waist circumference in adults: a meta-analysis. *Sleep* **38**, 1269-1276.

Sterne, J.A.C. and Egger, M. (2005) Regression methods to detect publication and other bias in meta-analysis. In: *Publication bias in meta-analysis: prevention, assessment and adjustments* (Rothstein, H.R., Sutton, A.J. and Borenstein, M.), pp. 99-110. England: John Wiley & Sons, Ltd.

Sutton, A.J. (2005) Evidence concerning the consequences of publication and related biases. In: *Publication bias in meta-analysis: prevention, assessment and adjustments* (Rothstein, H.R., Sutton, A.J. and Borenstein, M.), pp. 175-192. England: John Wiley & Sons, Ltd.

Terrin, N., Schmid, C.H., Lau, J. and Olkin I. (2003) Adjusting for publication bias in the presence of heterogeneity. *Stat. Med.* **22**, 2113–2126.

**Table and figure legends:**

**Figure S1** Summary effects (as natural logs, ln *R*) and 95% confidence intervals (CIs) for the influence of *CPA1* overexpression on shoot K^+^ concentration of plants exposed to NaCl. The impact of ten moderator variables on the magnitude of the treatment effect are portrayed (A-J). Category lists levels of each moderator. *n* is the number of studies contributing to each summary effect. *p* ≤0.05 indicates that the moderator level’s summary effect was significantly different than zero. Change refers to raw percentage increase in shoot K^+^ induced by overexpression of *CPA1*.

**Figure S2** Summary effects (as natural logs, ln *R*) and 95% confidence intervals (CIs) for the influence of *CPA1* overexpression on shoot K^+^/Na^+^ ratio of plants exposed to NaCl. The impact of nine moderator variables on the magnitude of the treatment effect are portrayed (A-I; gene number not analyzed due to insufficient studies). Category lists levels of each moderator. *n* is the number of studies contributing to each summary effect. *p* ≤0.05 indicates that the moderator level’s summary effect was significantly different than zero. Change refers to raw percentage increase in shoot K^+^/Na^+^ ratio induced by overexpression of *CPA1*.

**Figure S3** Summary effects (as natural logs, ln *R*) and 95% confidence intervals (CIs) for the influence of *CPA1* overexpression on shoot K^+^/Na^+^ ratio of plants exposed to NaCl. The impact of size moderator variables on the magnitude of the treatment effect are portrayed (A-F; some moderatos not analyzed due to insufficient studies). Category lists levels of each moderator. *n* is the number of studies contributing to each summary effect. *p* ≤0.05 indicates that the moderator level’s summary effect was significantly different than zero. Change refers to raw percentage increase in shoot K^+^/Na^+^ ratio induced by overexpression of *CPA1*.

**Table S1** Measures used in characterizing publication bias for each effect size

| Effect  sizes | Summary effect^1^ | | | Funnel^2^ | | Kendall^3^ | | Egger’s^4^ | | Orwin’s^5^ | | Duval & Tweedie^6^ | |
| --- | --- | --- | --- | --- | --- | --- | --- | --- | --- | --- | --- | --- | --- |
|  | N | ln*RR* | *p* | #  var | plot | tau | *p* | *β* | *p* | N | 5*k*+10 | adjusted | #  trim |
| Shoot Na^+^ | 114 | 0.166 | 0.024 | 7 | no | -0.08 | 0.21 | -1.03 | 0.03 | 4381 | 580 | 0.499 | 40 |
| Shoot K^+^ | 96 | 0.169 | 0.035 | 7 | no | -0.02 | 0.72 | 0.00 | 0.99 | 2384 | 490 | -0.032 | 36 |
| Root Na^+^ | 61 | -0.011 | 0.912 | 6 | no | -0.03 | 0.72 | -0.15 | 070 | ̶ | 315 | -0.222 | 18 |
| Root K^+^ | 57 | 0.272 | 0.008 | 6 | no | 0.06 | 0.52 | -0.02 | 0.95 | 3334 | 295 | 0.351 | 7 |
| Shoot Ca^++^ | 10 | 0.154 | 0.513 | 3 | yes | -0.67 | 0.01 | -1.36 | <0.01 | 1141 | 60 | 0.240 | 3 |
| Shoot K^+^/Na^+^ ratio | 25 | 0.466 | 0.009 | 3 | no | -0.09 | 0.53 | -0.85 | 0.29 | 644 | 135 | 0.618 | 6 |
| Root K^+^/Na^+^ ratio | 19 | 0.582 | 0.002 | 4 | yes | -0.08 | 0.62 | -0.87 | 0.22 | 306 | 105 | 0.733 | 4 |
| MDA (leaf) | 16 | -0.094 | 0.644 | 3 | no | -0.09 | 0.59 | 0.49 | 0.56 | ̶ | 90 | -0.094 | 0 |
| Leaf chlorophyll | 46 | 0.519 | <0.001 | 3 | no | -0.01 | 0.92 | 0.25 | 0.73 | 2047 | 240 | 0.624 | 7 |
| Shoot proline | 26 | 0.177 | 0.261 | 5 | no | -0.18 | 0.20 | -0.91 | 0.18 | 3555 | 140 | 0.387 | 7 |
| Shoot fresh weight | 33 | 0.284 | 0.028 | 5 | maybe | -0.13 | 0.28 | -0.99 | 0.04 | 8056 | 175 | 0.418 | 11 |
| Root fresh weight | 16 | -0.336 | 0.274 | 4 | maybe | 0.15 | 0.42 | 1.63 | 0.28 | 6324 | 90 | -0.877 | 5 |
| Total fresh weight | 29 | 0.298 | 0.014 | 7 | maybe | 0.24 | 0.26 | 1.05 | <0.01 | 7488 | 155 | 0.110 | 10 |
| Total dry weight | 30 | 0.476 | <0.001 | 6 | no | 0.05 | 0.71 | 0.67 | 0.22 | 3093 | 160 | 0.613 | 5 |
| Seedling height | 31 | 0.253 | 0.046 | 8 | no | 0.24 | 0.05 | 0.55 | 0.12 | 6590 | 165 | 0.265 | 1 |
| Root length | 43 | 0.298 | 0.005 | 6 | no | 0.14 | 0.17 | 0.64 | 0.14 | 1106 | 225 | 0.356 | 3 |
| Leaf REC | 21 | -0.386 | 0.042 | 3 | no | -0.11 | 0.47 | 0.27 | 0.57 | 6880 | 115 | -0.450 | 3 |
| Survuval test | 13 | -0.158 | 0.640 | 3 | no | -0.17 | 0.46 | -3.84 | 0.27 | 700 | 75 | -0.428 | 2 |
| Seed germination | 36 | 0.813 | 0.009 | 5 | no | 0.16 | 0.16 | -2.83 | 0.07 | 4807 | 190 | 1.582 | 13 |

^1^Summary effect: n=number of studies, ln*RR* = natural log of overall summary effect, *p*= probability that summary effect ≠ 0, #var = number of different variance values of studies comprising the summary effect

^2^Funnel plot appears asymmetrical

^3^Begg and Mazumdar Kendall rank correlation: tau = rank correlation coefficient (with continuity correction), two-tailed *p* = probability that study effect sizes are correlated with their sampling variances

^4^Egger’s linear regression: *β =* intercept of regression line, *p* = probability of significant asymmetry in study effect size/study size association. The regression runs through zero if the funnel plot is symmetrical. The size of the deviation of the intercept from the origin is a measure of asymmetry, with two-tailed *p* <0.05 indicating significant asymmetry (Sterne and Egger 2005).

^5^ Orwin’s failsafe statistics: N = number of missing studies needed to bring ln*RR* to a trivial point; 5*k*=10 is the classic (Rosenthal) number of missing studies that would be needed to be added to the analysis so that the summary effect would no longer be significant. We set the Orwin’s criterion (set point for trivial summary effect) and mean at 0.04 for each effect size. A ln*RR* of 0.04 represents a 4% treatment effect. Analysis not applicable when summary effect is nearer zero than criterion (denoted by dash).

^6^Duval and Tweedie trim and fill: adjusted summary effect after imputing missing studies using an iterative trim and fill procedure, #trim = number of studies imputed in the trim and fill exercise.
